# Exploring the driving forces of CO _2 _emissions in the European Union

**DOI:** 10.12688/openreseurope.20224.1

**Published:** 2025-05-12

**Authors:** Juan Cámara-Aceituno, Manuel Jesús Hermoso-Orzáez, Julio Terrados-Cepeda, Andrés Rivadeneira- Zambrano, Ángel Mena-Nieto, Antonio A. Golpe, Jose-Enrique Garcia-Ramos

**Affiliations:** 1 Department of Engineering Graphics, Design and Projects, University of Jaén, Jaén, Andalusia, 23701, Spain; 2 Department of Agroindustrial Processes, Technical University of Manabi, Chone, Manabí Province, Ecuador; 3 Department of Electrical and Thermal Engineering, Engineering Design and Projects, University of Huelva, Huelva, Andalusia, 21071, Spain; 4 Centre for Advanced Studies in Physics, Mathematics and Computation, University of Huelva, Huelva, Andalusia, 21071, Spain; 5 Department of Economics, University of Huelva, Huelva, Andalusia, 21071, Spain; 6 Department of Integrated Sciences, University of Huelva, Huelva, Andalusia, 21071, Spain; 7 Carlos I Institute of Theoretical and Computational Physics, University of Granada, Granada, Andalusia, 18071, Spain

**Keywords:** CO2 emissions, Kaya identity, European Union-27, LMDI decomposition, Tapio decoupling, Clubs of convergence, Clusters.

## Abstract

**Background:**

The analysis of the evolution of CO _2_ emissions of a given region is of key interest to understand the effect of past policies and to better design the future ones. The 27 European Union countries (EU) constitute a unique region for such a study because it has a strong common policy for reducing CO _2_ emissions and, therefore, it is of great interest to measure its influence.

**Methods:**

This study employed the logarithmic-mean Divisia index (LMDI) technique, an expanded version of the Kaya identity, the Tapio decoupling method, and convergence and cluster analysis. This study examined the driving forces behind CO _2_ emissions, including population, economic activity, energy intensity, and energy sources. The period under study is 1990–2021.

**Results:**

The results obtained for the 27 member states were diverse. However, some common patterns have emerged: economic activity is the primary driver of CO _2_ emissions, while energy intensity plays a crucial role in reducing emissions, even more than the contribution from renewable energies. The analysis reveals a consistent decline in recent years attributed to rigorous EU policies to meet the CO _2_ emissions target outlined in its Nationally Determined Contribution (NDC). Notably, countries with longer-standing EU memberships tend to exhibit more positive outcomes. Additionally, a study on the convergence of the 27 countries reveals the existence of several clusters and clubs of convergence.

**Conclusions:**

This study offers valuable insights for evaluating the energy and environmental policies of EU countries, serving as a valuable resource for energy policymakers worldwide.

## List of abbreviations

- act: economic activity.
- CO _2_: carbon dioxide.
- EU: European Union (twenty-seven member states).
- EI: energy intensity.
- GDP: Gross Domestic Product.
- GHG: greenhouse gases.
- Gtoe: Giga tonnes of oil equivalent.
- GtCO2: Giga tonne of CO _2_.
- int: energy intensity.
- IPCC: Intergovernmental Panel for Climate Change.
- kgCO2: kg of CO _2_.
- koe: kilograms of oil equivalent.
- LMDI: Logarithmic-mean Divisia index.
- mix: energy mix.
- Mtoe: Mega tonnes of oil equivalent.
- NDC: Nationally Determined Contribution.
- OECD: Organisation for Economic Cooperation and Development.
- PC: Principal Component.
- PCA: Principal Component Analysis.
- pop: population.
- tCO2: tonnes of CO _2_.
- toe: tonnes of oil equivalent.
- UNFCCC: United Nations Framework Convention on Climate Change.
- USD: 2010 constant international dollar.

## 1. Introduction

Anthropogenic CO _2_ emissions are currently in the spotlight, and for a significant fraction of the population is considered a major concern because of their causal relationship with global warming and Climate Change. The most recent Intergovernmental Panel for Climate Change (IPCC) assessment report ^1, 2^ underscores a clear and almost irrefutable correlation between human actions and the steady increase in Earth’s mean temperature over the past century. Indeed, the temperature has increased by approximately 1 ^*◦*^C in the last century. CO _2_ emissions appear to be the origin of the connection between global warming and human activity. However, it is worth not forgetting other greenhouse gases (GHG) such as CH _4_, NO _*x*_, or hydrofluorocarbons (HFCs) and perfluorocarbons (PFCs), which largely contribute to the greenhouse effect ^3^.

Many factors influence the amount of CO _2_ emissions, including economic development, population growth, used technology, institutional structures, international trade, and lifestyle. Therefore, it is crucial to identify the main driving forces that modulate CO _2_ emissions and present them to policymakers. This will allow them to define appropriate future policies for CO _2_ emissions. As a matter of fact, CO _2_ emissions are caused by economic activity through energy use. Indeed, this causal relationship was proposed by K. Kaya in the 1990s ^4– 6^, and shortly thereafter, the term *Kaya identity* was introduced. The Kaya identity expresses CO _2_ emissions as a function of population, GDP per capita, energy intensity, i.e., energy use per unit of GDP, and emission factors, i.e. CO _2_ emissions per unit of energy. It has been used extensively to estimate CO _2_ emissions in the medium and short term within the framework of climate scenarios.

This study focuses on a detailed description of the evolution of the different contributions to CO _2_ emissions as a function of time for the period 1990–2021 for the European Union (EU ^1^). The EU, as a whole, represents “only” 10% of global CO _2_ emissions. However, it is a region in which different countries carry out strongly coordinated efforts to reduce them. Moreover, it is probably the largest region in the world with the most stringent National Determined Contribution (NDC) ^7^. Therefore, the EU could be regarded from the outside as an example to be followed or as a test bed for new policies promoting the reduction of CO _2_ emissions; therefore, it will be of great interest to understand the effect of EU policy on past emissions.

The first EU NDC was approved in March 2015 and established a 40% reduction in GHG emissions by 2030 compared to 1990, but the target was increased to 55% in the 2023 update. The EU NDC affects and will affect most economic sectors, namely, energy, industrial processes and product use, agriculture, waste, and land use. This implies that it is crucial for policymakers to have a clear view of the influence of the different driving forces of CO _2_ emissions, and to define the most appropriate policies to slow down the increase in CO _2_ emissions.

In this work, we use an enlarged version of the Kaya identity, in which the energy is disaggregated by the type of fuel, namely, fossil (solid, liquid, or gas) or renewable, considering four types of energy sources. In previous works ^8– 13^, we used a more refined grain disaggregation; however, in this case, owing to the large number of countries considered, a certain simplification is considered to perform global analyses in a better way. To analyze in depth the temporal evolution of the CO _2_ driving forces, we used the logarithmic mean Divisia index (LMDI) ^14^ technique together with the Tapio decoupling analysis ^15^. Currently, the LMDI method has become a key tool for determining the nature of the factors influencing energy consumption changes ^16^. Additional information will be obtained through the Tapio decoupling analysis, particularly its time evolution, providing an even more straightforward way to compare different countries. Moreover, to have a clearer view of how the situation of different countries has evolved, we will consider a convergence study ^17^, which will allow us to establish groups of countries with similar behavior that move into a common end. Cluster analysis will also be considered, using the K-means algorithm ^18^ to complement the convergence study, to separate the countries into groups with a similar global trend. The study period analyzed is especially relevant because it started in 1990, which is the reference date for the EU NDC. Moreover, it goes beyond the period of the COVID-19 outbreak.

The above paragraph presents the literature gap covered in this work, namely, the LMDI time evolution analysis of the CO _2_ driving forces, its Tapio´s decoupling analysis, also presented as a function of time, and convergence and cluster studies. All these analyses will be performed for the 27 EU members, which will provide critical information for policymakers in a very compact way. To the best of our knowledge, such a comprehensive analysis using these five methods for the EU has not been published before.

The remainder of this paper is organized as follows. Section 2 reviews the relevant literature concerning the analysis of CO _2_ emissions in the EU; Section 3 presents the area under study; in Section 4, the methodology used is sketched; and Section 5 demonstrates the primary outcome of this study and the discussion. Finally, Section 6 presents the conclusions and policy implications.

## 2. Literature review

The literature on the driving forces of CO _2_ emissions, particularly their connection with economic development and energy consumption, is extensive. In this section, we will concentrate on those recent works that apply methodologies similar to those used in this work and are akin to the set of countries considered in this analysis. The Kaya identity is jointly applied with the LMDI decomposition analysis, Tapio decoupling analysis, and clubs of convergence and cluster studies.

In 19, the authors studied the case of the EU together with China, India, and the USA. The authors conducted a decomposition analysis using multiple models for various scenarios between 2000 and 2100. They identified the assumptions and model characteristics that lead to different decomposition results in moderate and stringent climate policy scenarios. In 20, 21, the authors performed an LMDI study for the EU from 2001 to 2010 using the Kaya identity and decomposing the emissions into contributions from the population, GDP per capita, energy intensity, energy mix, and CO _2_ intensity. They concluded that changes in the energy mix strongly impacted emissions. Another relevant work in the EU is 22, where the authors also performed an LMDI analysis for CO _2_ emissions for the member states of the EU during the period 1990–2010, disaggregating CO _2_ intensity, fossil fuel consumption relative to total energy, energy intensity, GDP over imported petroleum products, imported petroleum products per capita, and population. The authors detected different adaptation velocities for the Kyoto targets. Besides, they found that energy intensity and petroleum imports were the main driving forces in the increase in CO _2_ emissions. In 23, the authors performed a decomposition analysis of the driving forces of CO _2_ emissions in the EU-15, namely Austria, Belgium, Denmark, Finland, France, Germany, Greece, Ireland, Italy, Luxembourg, Netherlands, Portugal, Spain, Sweden, and the UK. 24 is devoted to the decomposition analysis of the driving forces of CO _2_ emissions in the EU power sector. They considered four different periods to disentangle, which had a more significant influence on the CO _2_ emissions. They found essential variations owing to the activation of the Kyoto Protocol. On the other hand, 25 focused on the drivers of CO _2_ emissions and other GHGs in the Baltic states of Lithuania, Latvia, and Estonia. They showed that the emissions decreased by 55% from 1990 to 2012. This achievement is mainly due to better energy efficiency and more renewable energy use. 26 studies how economic growth, renewable energy, and natural resources influence CO _2_ emissions in the five largest economies of the EU, namely, Germany, France, Italy, Spain, and the United Kingdom. The ^27^ study analyzes the relationship between carbon emissions, real income, energy consumption, and tourism for a panel of the EU and candidate countries from 1995 to 2011. Research ^28^ has focused on the decomposition of energy and CO _2_ intensity in the EU. Their results showed that the total energy use decreased because the region moved to less energy-intensive industries and improved sectoral energy efficiency. CO _2_ emissions were also reduced owing to the decrease in energy consumption and the emission factor. Another relevant study focused on Greece ^29^. The authors also applied the LMDI and arithmetic mean Divisia index to study CO _2_ emissions from 1990 to 2002. They showed that the income effect is the leading cause of the increase in CO _2_ emissions.

Concerning the use of the Tapio decoupling analysis, it is worth mentioning the study ^30^, which constitutes an update of the work ^24^, where the authors examined the CO _2_ emissions of electricity generation in the EU using the Tapio decoupling analysis after an LMDI study. The authors separated the studied period, 2000–2018, into three subperiods to analyze the time evolution of the decoupling factor. The main conclusion of this study is that most European countries moved into a strong decoupling phase in the third period. In 31, the authors also conducted an LMDI analysis followed by a Tapio decoupling study for the EU in the period 1996–2017, paying particular attention to the effect of “outsourcing”, i.e., the transfer of part of the production outside the EU. The main conclusion is that travel into a strong decoupling phase is faster due to “outsourcing.” Among the latest studies on the trend of CO _2_ emissions in the EU, a recent study ^32^ used the LMDI analysis and Tapio’s decoupling method between 1995 and 2019 by decomposing emissions into seven factors. The main conclusion of their study was that factors such as energy efficiency and the renewable mix were the drivers of the decrease in emissions. Furthermore, application of the Tapio method revealed that the EU achieved significant decoupling. However, this study also identified clear differences among countries.

Convergence analysis has garnered considerable attention in recent years, serving as a tool for examining whether countries, regions, or states achieve the same or different steady states in specific areas. Numerous studies have employed the club convergence methodology developed in 17, 33 to test this hypothesis. This approach identifies groups of countries (clubs) converging toward distinct steady states, rather than simply assuming a single common state for all. Notably, this methodology has been applied to investigate convergence patterns in South America ^34^, in European and OECD countries, utilizing diverse environmental variables such as CO _2_ emissions, in total levels ^35, 36^ or per capita ^37^, GHG emissions, overall emissions ^38^, sectoral differences ^39, 40^, carbon intensity ^41^, and renewable energy sources ^42, 43^, among others.

## 3. Overview of the study area

The EU is a large region with a surface area of 4 million km ^2^ and population of 447 million inhabitants ^44^ in 2021. This number was relatively stable, with a modest increase from 1990 to 2021 (approximately 6 *.*29%). In 2021, the EU is at the same time one of the wealthiest regions in the world with a GDP of more than 14.69 trillion USD ^2^, slightly below that of China (15 *.*85 trillion USD) and significantly smaller than the USA (20 *.*52 trillion USD). Additionally, its per capita GDP is around 32857 USD (in 2021), much higher than that of China (11223 USD) and significantly lower than that of the USA (61829 USD). GDP increased by 35 % from 1995 to 2008, followed by a substantial dip of 4% from 2008 to 2009. However, from 2012 onwards, GDP has steadily increased, except for the sudden drop in 2019–2020 due to the COVID-19 outbreak (6% reduction according to 44). The sectoral structure of the EU is such that 1 *.*8% of GDP corresponds to the primary sector (agriculture, forestry, and fishing), 25 *.*5% to the secondary sector (manufacturing, mining, quarrying, electricity, gas, water supply, and construction), and 72 *.*7% to the tertiary sector (services, trade, residential, and transportation) ^44^.

The EU, as a whole, is one of the largest energy consumers in the world. Its energy consumption per capita reached a maximum of 3617 koe ^3^ in 2004, although this quantity decreased to 3207 koe in 2016. During the past decades, primary energy production from fossil fuels and nuclear energy has decreased. In particular, petroleum products presented a decrease of 52%, whereas gas production decreased by 43%. However, there was a significant increase (73%) in the production of renewable energy during the same period ^45^. The efficiency in the use of energy, defined as the energy intensity (energy over GDP), has improved over the last decades, passing from 130 koe/1000 USD to 80 koe/1000 USD, which strongly contributed to the reduction of the energy consumption of the region.

The EU is a significant contributor to CO _2_ emissions, with a total quantity of about 3780 Mton of CO _2_ in 1990 and of around 2630 Mton of CO _2_ in 2020, which corresponds to 8 *.*98 ton per capita in 1990 and to 5 *.*88 ton per capita in 2020, to be compared with the USA 19 *.*4 ton per capita in 1990 and 13 ton per capita in 2020, or with China 1 *.*91 ton per capita in 1990 and 7 *.*75 ton per capita in 2020. The considerable reduction in emissions per capita in the EU (and USA) clearly shows the importance of this issue for European policymakers and society in general.

To summarize the characteristics discussed above, the EU presents unique dynamics that makes it a crucial region for studying the impacts and solutions of CO _2_ emissions. This is because, although its GDP is lower than that of China and the USA, it stands out for having a proactive and ambitious climate change policy, leading global initiatives such as the Paris Agreement, while setting binding targets for carbon emission reductions.

It is also worth highlighting the potential of the region for its focus on energy efficiency, decarbonization (with policies such as the European Green Deal that aims for climate neutrality by 2050), and sustainability (transition toward an energy model based on renewable energies) that make the EU a global benchmark in the study of GHG mitigation. Moreover, while China and the USA are more dependent on economic growth based on carbon-intensive industries, the EU shows a partial decoupling of economic growth from carbon emissions through the observed decline in per capita emissions. This makes the region a key case for understanding how countries can maintain economic growth while reducing their environmental impacts. The combination of these factors, coupled with their role as one of the world’s most integrated economies, makes the EU a model region for studying the driving forces of carbon emissions and mitigation strategies at the global level.

## 4. Material and methods

### 4.1 The Kaya identity

The key element for the calculation of CO _2_ emissions is the variation in the Kaya identity ^4– 6^, where the energy is disaggregated. Hence, CO _2_ emissions from industry and other energy uses were categorized into five factors: population, GDP per capita, energy intensity, energy mix, and CO _2_ emissions. Therefore, the CO _2_ emissions can be written as


$$C=\sum_{j}C_{j}=\sum_{j}P\frac{Q}{P}\frac{E}{Q}\frac{E_{j}}{E}\frac{C_{j}}{E_{j}}=P\cdot q\cdot EI\cdot \sum_{j}M_{j}\cdot U_{j},\;(1)$$

where *C* is the total CO _2_ emissions of a given country in the EU (in a given year), *C _j_* is the CO _2_ emissions arising from fuel type *j* (the index *j* runs over four kinds of energy sources), *P* is the population, *Q* is the GDP of the country, *q* is the GDP per capita, *E* is the energy consumption, *E _j_* is the consumption of fuel of type *j*, *EI* $\left(\frac{E}{Q}\right)$ refers to the energy intensity, the energy mix is given by *M _j_* $\left(\frac{E_{j}}{E}\right)$ , and the CO _2_ emission factor is *U _j_* $(\frac{C_{j}}{E_{j}}).$

### 4.2 The Logarithmic Mean Divisia Index (LMDI)

The LMDI method provides a clever way of separating the different contributions to CO _2_ emissions appearing in the Kaya identity, namely, population ( *pop*), activity ( *act*), energy intensity ( *int*), energy mix ( *mix*), and emission factor ( *emission*). However, this study assumes no variation owing to the emission factor. The LMDI corresponds to the sum of the relative changes weighted appropriately and uses the Divisia index concept proposed by Divisia in the 1920s. The term “Logarithmic Mean Divisia Index” was coined in 46, and since then, the use of the LMDI has rapidly grown thanks to the works ^47– 50^.

In short, the LMDI method aims to express the value of a given aggregated variable in a given year, *t*, with respect to a reference one, 0, CO _2_ emissions in our case, in terms of the sum of the contributions of different driving forces. LMDI has two flavors: additive and multiplicative. The additive is written as


$$\Delta C(t)=C(t)-C(0)=\Delta C_{pop}(t)+\Delta C_{act}(t)+\Delta C_{int}(t)+\Delta C_{mix}(t),\;(2)$$

where Δ *C _driver_* = *C _driver_* ( *t*) – *C _driver_* ( *0*) and driver stands for *pop, act, int, mix*, and finally


$$\;\Delta C_{pop}(t)=\sum_{j}\frac{C_{j}(t)-C_{j}(0)}{lnC_{j}(t)-lnC_{j}(0)}ln\frac{P(t)}{P(0)},\;(3)$$

$$\;\Delta C_{act}(t)=\sum_{j}\frac{C_{j}(t)-C_{j}(0)}{lnC_{j}(t)-lnC_{j}(0)}ln\frac{q(t)}{q(0)},\;(4)$$

$$\;\Delta C_{int}(t)=\sum_{j}\frac{C_{j}(t)-C_{j}(0)}{lnC_{j}(t)-lnC_{j}(0)}ln\frac{EI(t)}{EI(0)},\;(5)$$

$$\;\Delta C_{mix}(t)=\sum_{j}\frac{C_{j}(t)-C_{j}(0)}{lnC_{j}(t)-lnC_{j}(0)}ln\frac{M_{j}(t)}{M_{j}(0)}.\;(6)$$

It is also possible to perform the decomposition in a multiplicative way obtaining,


$$\;D(t)=C(t)/C(0)=D_{pop}(t)\cdot D_{act}(t)\cdot D_{int}(t)\cdot D_{mix}(t),\;(7)$$

where *D _driver_* ( *t*) = *C _driver_* ( *t*)/ *C _driver_* (0). These factors can be readily calculated using Equation (3) – Equation (6), as follows:


$$\;D_{driver}(t)=exp\left(\frac{\Delta C_{driver}}{\frac{C(t)-C(0)}{lnC(t)-lnC(0)}}\right).\;(8)$$

### 4.3 Tapio decoupling method

The “decoupling” theory was introduced by Tapio in the seminal works ^15, 51^ in the framework of the analysis of CO _2_ emissions due to transport and their relationship with GDP. Its main advantage is its simplicity in defining how strongly emissions and GDP are connected. Moreover, it can be easily connected to additive LMDI results. In short, Tapio decoupling is defined in terms of elasticity, written as:


$$\varepsilon =\frac{\Delta C/C(0)}{\Delta Q/Q(0)}=\frac{\Delta C_{pop}+\Delta C_{act}+\Delta C_{int}+\Delta C_{mix}}{Q(t)-Q(0)}\times \frac{Q(0)}{C(0)},\;(9)$$

**Figure 1.**
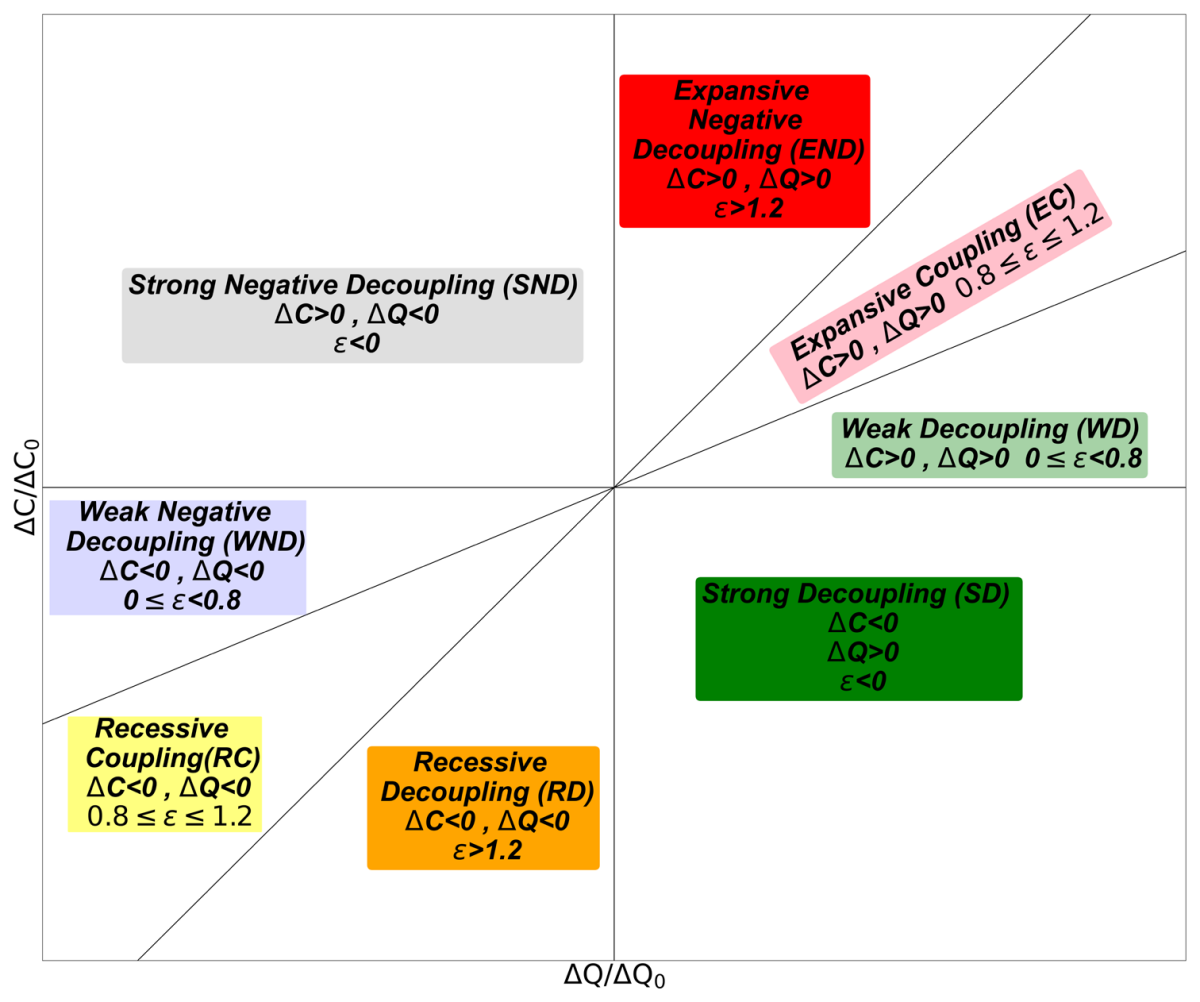
Tapio decoupling index classification. Eight different regions are defined. WD: weak decoupling, EC: expansive coupling, END: expansive negative decoupling, SND: strong negative decoupling, WND: weak negative decoupling, RC: recessive coupling, RD: recessive decoupling, and SD: strong decoupling.

According to 15, 51, it is possible to differentiate eight situations depending on the value of *ε* and the sign of the growth rate of the GDP and the CO _2_ emission, as depicted in Figure 1. These two growth rates can be coupled, decoupled, or negatively decoupled, and different areas are defined considering the line of elasticity *ε* = 1 plus and minus 20% as a reference. Hence, the areas with different signs of growth rates will correspond to Strong Decoupling (SD) for a positive GDP growth rate or Strong Negative Decoupling (SND) for a negative GDP growth rate. For both positive growth rates, one has Weak Decoupling ( *ε <* 0 *.*8) (WD), Expansive Coupling (0 *.*8 *< ε <* 1 *.*2) (EC) and Expansive Negative Decoupling ( *ε >* 1 *.*2) (END). For both negative growth rates, the regions are Weak Negative Decoupling ( *ε <* 0 *.*8) (WND), Recessive Coupling (0 *.*8 *< ε <* 1 *.*2) (RC), and Recessive Decoupling ( *ε >* 1 *.*2) (RD). Clearly, the situations in which the connection between GDP and CO _2_ emissions is less affecting the environment correspond to Strong Decoupling, Weak Decoupling, and Recessive Decoupling because a positive GDP growth rate corresponds to a low or negative CO _2_ growth rate. In contrast, a negative GDP growth rate maximizes the reduction of CO _2_ emissions.

### 4.4 Convergence analysis

The procedure developed by Phillips and Sul ^17, 33^ was employed in this study to assess the convergence dynamics of the Kaya components in a panel of EU countries and to detect any convergence clubs across them. This procedure aims to identify groups of countries within a panel that exhibit similar behavior in their convergence patterns, even in the absence of total convergence. This procedure may detect the presence of several convergence clubs and permit certain countries to diverge.

The starting point is given by Equation (10), where each Kaya component, *KY _i_*_*t*_, is quantified for a given state *i* and time *t* in terms of the parameter *δ*_*i*_, which represents the average distance, *µ*_*t*_, which refers to the common systematic part, and, *ε _it_* which corresponds to the error term,


$$\;KY_{it}=\delta_{i}\mu_{t}+\varepsilon_{it}\;(10)$$

where *a _it_* and *x _it_* represent systematic and transitory components, respectively. Hence, the following expression can be obtained:


$$\;KY_{it}=a_{it}+x_{it}\;(11)$$

Combining both expressions Phillips and Sul ^17^ obtain,


$$\;KY_{it}=\left(\frac{a_{it}+x_{it}}{\mu_{t}}\right)\mu_{t}=\delta_{it}\mu_{t},\;\forall i,t\;(12)$$

where *δ _it_* and *µ _t_* reflect time-dependent idiosyncratic and common trend components, respectively. As a matter of fact, *δ _it_* gives evidence of the transition path of each individual to the common steady-state path, which is represented by *µ*_*t*_. To remove the common component, therefore separating the idiosyncratic component, and to check whether *δ _i_* converges to a constant value, *δ*, Phillips and Sul ^17^ proposed achieving ratios to describe a relative transition parameter, *h _i_*_*t*_:


$$\;h_{it}=\left(\frac{KY_{it}}{N^{-1}\sum_{i=1}^{N}KY_{it}}\right)=\frac{\delta_{it}}{N^{-1}\sum_{i=1}^{N}\delta_{it}},\;(13)$$

where *h _it_* estimates the evolution of the variable of interest for country *i,* relative to the panel average. In the case of ultimate convergence among individuals, *h _it_* → 1 and *H _it_* → 0 as *t* → ∞, where $H_{it}=N^{-1}\sum_{i=1}^{N}{\left(h_{it}-1\right)}^{2}$ reflects the cross-sectional change in the relative transition growth path. The null hypothesis of Phillips and Sul’s ^17^ test can be expressed within a semiparametric model for *δ _i_*_*t*_:


$$\;\delta_{it}=\delta_{i}+\frac{\sigma_{i}\xi_{it}}{L(t)t^{\alpha }},\;with\;t\geq 1,\;\sigma_{i}\geq 0\;\forall i,\;(14)$$

where *δi* is fixed, *ξ _it_* is i.i.d. (0,1) across *i* but weakly dependent on *t*, *L*( *t*) is a slowly varying function that increases and diverges at infinity, and *α* corresponds to the decay rate, such that *δ _it_* converges to *δ _i_* when *α ≥* 0. Thus, the null and alternative hypotheses for convergence are


$$H_{0}:\delta_{it}=\delta_{i}\;and\;\alpha \geq 0$$

$$H_{1}:\delta_{it}\neq \delta_{i},\;\forall i\;or\;\alpha <0$$

The default hypothesis of convergence can be tested following the regression model,


$$\;log\left(\frac{H_{1}}{H_{t}}\right)-2logL(t)=\hat{c}+\hat{b}\;log(t)+u_{t}\;(15)$$

Full panel convergence exists and is statistically significant when $\hat{b}$ *≥* 0. Otherwise, divergence occurs in the panel. Hence, higher values of $\hat{b}$ imply faster convergence. Finally, rejection of the null hypothesis suggests the absence of full convergence in the panel but allows the possibility of different subgroups (convergence clubs) with diverse convergent patterns.

### 4.5 Cluster and principal component analysis

The goal of performing cluster analysis is to consider all the information of the different LMDI components to analyze the similarities between the different members and separate them into groups and clusters with a similar trend. The chosen method was the K-means method proposed in 18. K-means is a widely used clustering algorithm in which given a set of *N* samples $\vec{X}=\{{\vec{x}}_{I},\;...\;,{\vec{x}}_{n}\}$ and several clusters *K*, their centroids $\vec{C}=\{{\vec{c}}_{1},\;...\;,{\vec{c}}_{K}\}$ are computed by minimizing this cost function:


$$\;f(\vec{C})=\sum_{i}^{N}\;\sum_{j}^{k}w_{i,j}{\left(\left|\left|{\vec{x}}_{i}-{\vec{c}}_{j}\right|\right|\right)}^{2},\;(15)$$

where *w _i,j_* ∈ {0, 1} is the partition matrix that defines the clusters. Hence, if *i* is assigned to cluster *k*, *w _i,k_* = 1; otherwise, it is 0. Note that $\vec{x}$ _i_ *and* $\vec{c}$ _j_ correspond to the multidimensional vectors.

Once fixed the number of clusters, K, the initial centroids are initialized, either randomly or through some initialization function, and each sample is assigned to the closest centroid. The mean position of the samples corresponding to a given cluster defines the centroid position of each cluster. This process was repeated until the cost function was minimized.

Because the vector dimension that characterizes the sample members $\vec{x}$ _i_ can be very large, it is impossible to obtain a pictorial view of the sample itself. In other cases, such a high dimension can affect the performance of data analysis, such as K-means in our case. Therefore, it is beneficial to perform dimensionality reduction. One of the most popular is the Principal Component Analysis (PCA) ^52^. The PCA makes a linear dataset transformation so that the transformed components are statistically uncorrelated. The first principal component (PC) can also be defined as the direction with the largest variance. The second PC is perpendicular to the first, maximizing variance, and so on. Therefore, the eigenvectors of the covariance matrix of the data are PC’s. PCA was used to reduce dimensionality, considering only the PCs with the largest variance. Indeed, in our case, the two PCs were sufficient to properly characterize the dataset.

### 4.6 Sources of data

The data considered in this work were obtained from the official databases of Eurostat ^45^, the World Bank ^44^, the IPCC ^53^, and the United States Environmental Protection Agency (EPA) ^3^.

The units for energy are the oil equivalent (koe, toe, Mtoe, or Gtoe), and GDP is the 2010 constant US dollar (USD). The International System of Units was employed for the remainder. The carbon-free emission sources considered were solid biofuel, solar, wind, nuclear, and hydroelectric energy.

For simplicity, we consider only three types of fossil fuels that correspond to aggregate quantities. Their emission factors were 3, 54 U _solid_ = 4 *.*082 kgCO _2_/koe, U _liquid_ = 3 *.*069 kgCO _2_/koe, and U _gas_= 2 *.*349 kgCO _2_/koe, while the emission factor for renewable energy was assumed to be zero.

The period considered starts in 1990 and finishes in 2021. This is a very relevant period because, on the one hand, 1990 corresponds to the reference year for the EU NDC ^7^, and one can observe the effect of the “subprime” mortgage crisis as well as the impact of the COVID-19 outbreak.

## 5. Empirical results and discussion

### 5.1 Decomposition analysis

**Figure 2.**
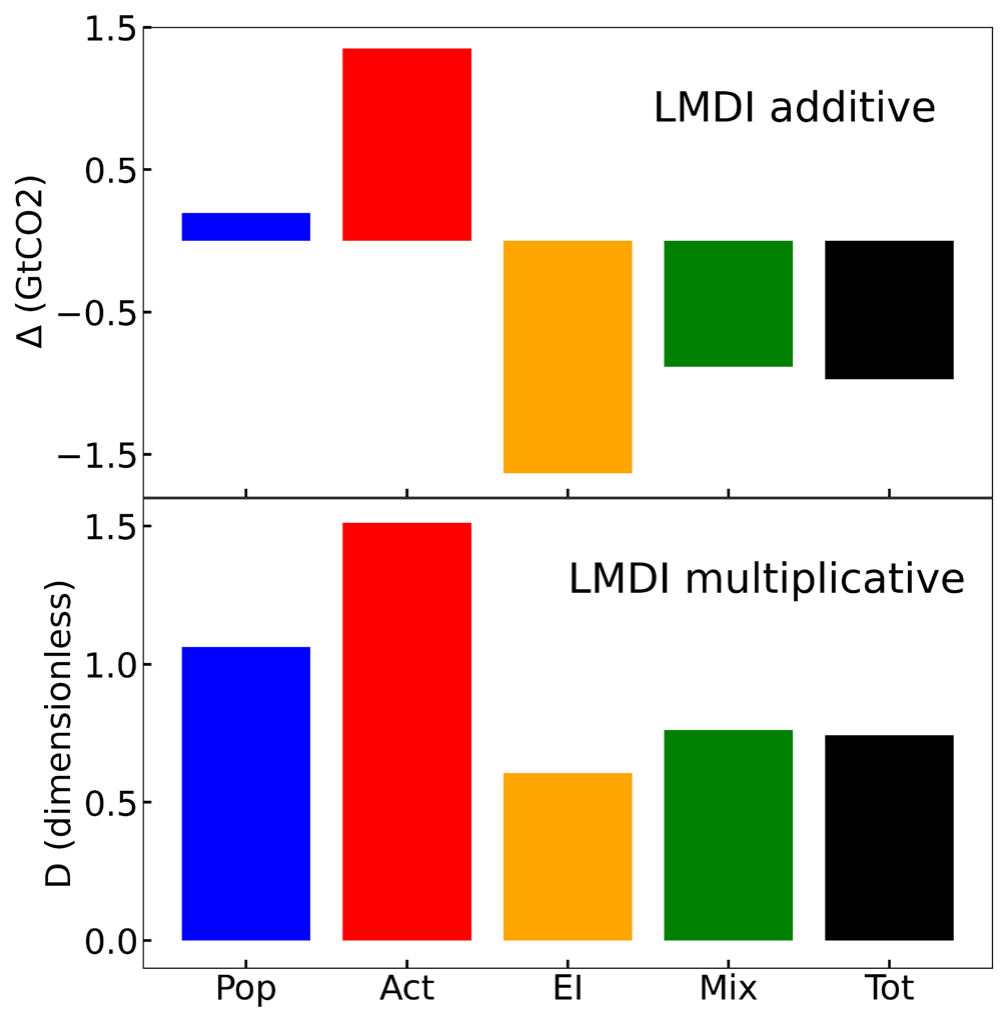
Additive and multiplicative LMDI decomposition for the whole EU and the final year 2021.

The LMDI provides a clever way to decompose the increase in CO _2_ emissions in a given period into its driving forces. The result for 2021, referring to 1990 for the whole EU, is depicted in Figure 2, in both the additive and multiplicative way. The first striking point is that the emissions were reduced by 26% (−0.97 GtCO _2_) in 2021 with respect to 1990, which is in line with the target established in the EU NDC, which corresponds to a reduction of 55% by 2030. The main contributor to this reduction was the energy intensity (−40%, −1.63 GtCO _2_) and, at a smaller pace, the energy mix (−14%, −0.89 GtCO _2_). On the contrary, the activity term is by far the largest contributor to the increase in CO _2_ emissions (51%, 1.35 GtCO _2_), followed by population (6%, 0.2 GtCO _2_).

**Figure 3.**
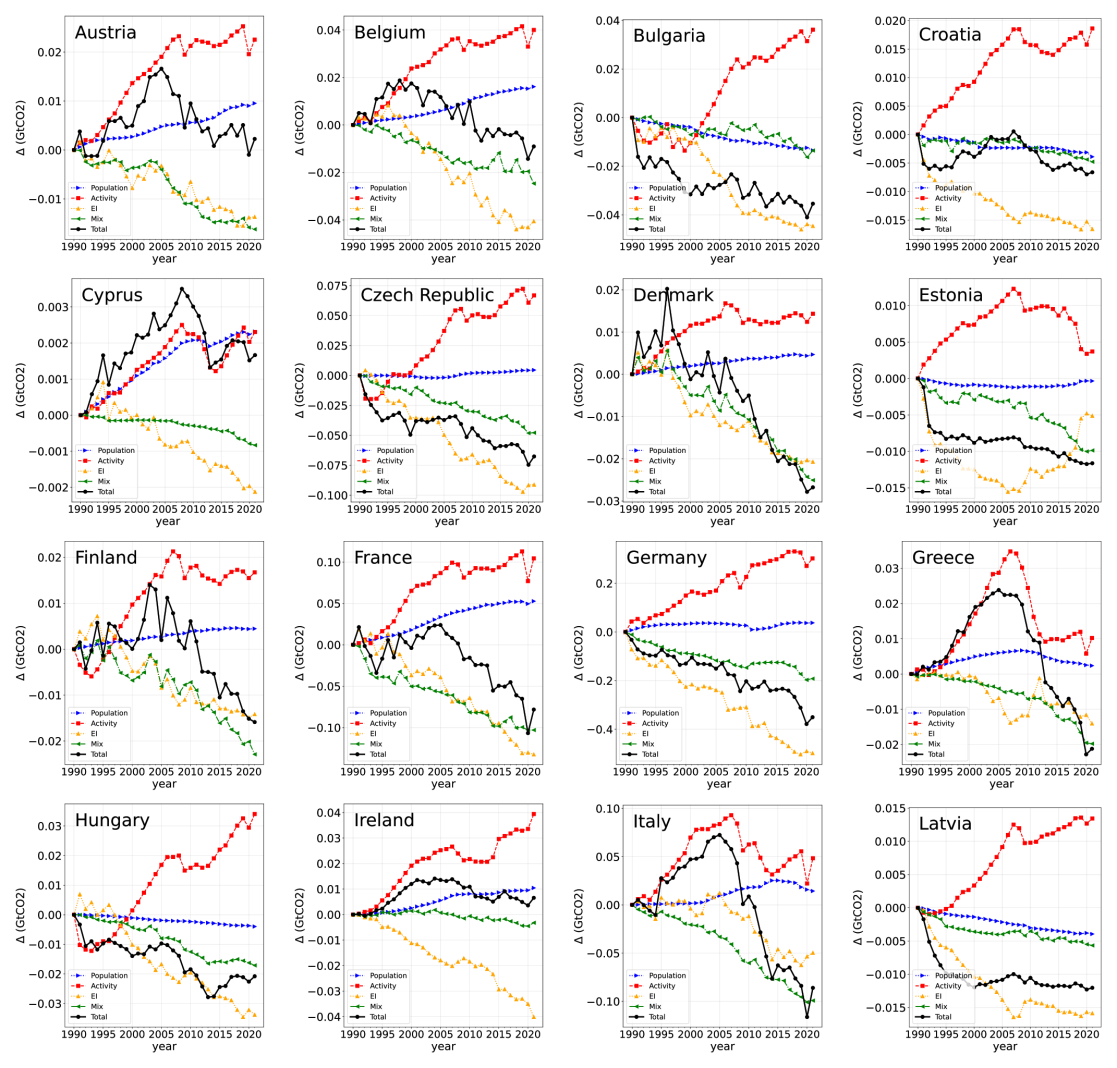
Additive LMDI decomposition of the 27 countries from 1990 to 2021.

**Figure 4.**
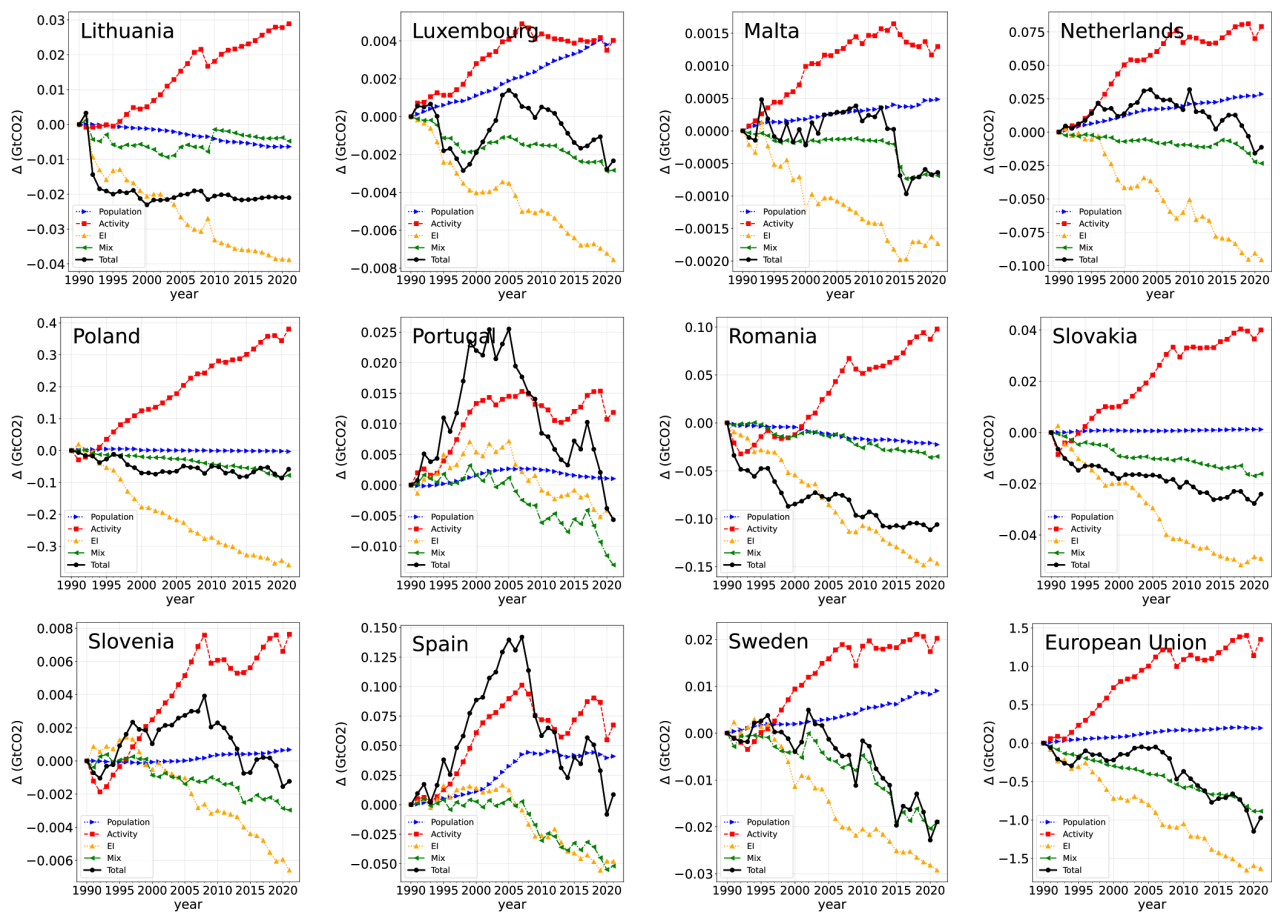
Same as Figure 3.

The former analysis lacks information about the evolution of time and the different behaviors of different countries. In Figure 3– Figure 4, the evolution of the LMDI components for all EU countries, including the EU as a whole. The multiplicative LMDI components are presented in Figs. A1-A2 in 55. As global conclusions from these figures, one can extract, on the one hand, that the energy intensity is the main driving force for reducing CO _2_ emissions, followed by the energy mix. However, the term activity is the driving force that contributes the most to the increase in CO _2_ emissions. In fact, a sudden drop can be easily observed during the 2008 economic crisis and the COVID-19 outbreak in 2020. Finally, the population term has a small influence in general, but in certain countries, its effect is not negligible, such as Luxembourg, Spain, and Sweden.

We describe the trend of the driving forces of CO _2_ emissions for all EU countries in an abridged manner.

In Austria (AU), emissions reached a maximum around 2005 with no net reduction in 2021; the energy mix and energy intensity terms contribute equally to reducing emissions; the population presents a positive contribution throughout, and the activity term shows certain stabilization in the last decade.

In Belgium (BE), emissions reached a maximum around 2000 with a net reduction in 2021. The term that presents the largest negative contribution is energy intensity, followed by energy mix. The population shows a positive slope, whereas the activity term is always positive, with a specific stabilization in the final period.

In Bulgaria (BG), the emissions show a reduction all the way, although with a somewhat erratic trend; population and energy mix present a modest similar negative contribution during the whole period, energy intensity provides the most considerable reduction, and finally, the activity term shows a negative contribution to the emissions at the beginning of the period followed by a rapid increase and stabilization in the final period.

In Croatia (HZ), the population, energy mix, and energy intensity terms behave as in Bulgaria; the activity term shows a rapid increase until 2010 and later a stabilization; finally, the total emissions show certain oscillations with a net reduction at the end of the period.

In Cyprus (CY), the emissions show a net increase during the whole period, with a maximum around 2010; population and activity terms contribute almost the same to the increase in emissions; the reduction coming from the energy mix term is almost negligible, except at the end of the period, and the energy intensity term presents a clear negative slope.

In the Czech Republic (CZ), total emissions continuously decrease as the energy mix and energy intensity contribute, and the effect of population contribution is almost unnoticeable. At the same time, the activity term shows a certain decrease at the beginning of the period, followed by a rapid increase, with final stabilization.

In Denmark (DK), total emissions, energy mix, and energy intensity decreased intensely throughout the period, with a small contribution from the population term. The activity term has a relatively constant contribution at all times, except at the beginning of the period when an upslope trend was observed.

In Estonia (EE), a somewhat anomalous trend is observed, with the energy intensity mix showing a parabolic shape. The activity term presents a maximum around 2008; the energy mix term shows a modest but constantly decreasing trend, while the total emissions show a rapid decrease at the beginning of the period, with an almost constant contribution at the middle of the period, with an additional modest reduction at the end.

In Finland (FI), energy mix and energy intensity contribution present a similar decreasing trend; population contribution is small but positive all the time, activity term shows an abrupt drop, then a rapid increase, and finally a stabilization, with the total emissions showing a global downward sloping trend.

In France (FR), the energy mix and energy intensity terms have a strong down-sloping contribution, population presents a sizable positive contribution, the activity term is the main positive contribution with a specific stabilization at the end of the period, and the total emissions show a consistent decrease at all times.

In Germany (DE), which is the leading actor that determines the EU trend, energy intensity and energy mix contributions present a clear and smooth negative trend, being more intense in the first case. The contribution coming from the population is almost negligible, and the activity contribution presents a relatively smooth up sloping trend, with a clear and smooth decrease in the total emissions.

In Greece (GR), total emissions increased rapidly, reaching a maximum around 2008 and then rapidly decreasing, followed by a similar trend, although it stabilized at the end of the period; the population contribution, energy mix, and energy intensity terms showed a global downslope trend.

In Hungary (HU), the energy mix and energy intensity contributions show a downsloping trend being steeper with the latter; the population contribution is almost unnoticeable but negative; the activity contribution dropped at the beginning of the period and then increased constantly for the rest of it, while the total emissions decreased during the entire period.

In Ireland (IE), the contribution to energy intensity is by far the most negative term. In contrast, the contribution of the energy mix is negative but modest. The population contribution was also small and positive, and the activity contribution showed a constant up-sloping trend. Finally, the total emissions at the end of the period were almost equal to the initial emissions, with a maximum around 2005.

In Italy (IT), the observed trends are essentially the same as in Greece.

In Latvia (LV), population and energy mix contributions are smooth and modest; energy intensity presents a strong negative contribution throughout the period, the activity term shows a rapid increase throughout the period, and total emissions drop rapidly at the beginning of the period and remain constant thereafter.

In Lithuania (LT), the observed trends were mostly equal to those in Latvia.

In Luxembourg (LU), the energy intensity contribution is strong and negative all the time; the energy mix contribution is also negative, but to a lesser extent; the population contribution shows an up-sloping trend, with a noticeable contribution; the activity term contribution increases continuously until the mid-period and then stabilizes; finally, the total emissions at the end of the period present a net reduction, but the trend is not smooth.

In Malta (MT), the energy intensity term presents a clear down-sloping trend, the effect of the population is small but positive, the activity term shows an up-sloping trend until 2015 with a final stabilization, and finally, the energy mix contribution and total emissions present a null value until 2015, where a step discontinuity is observed, leading to a net decrease in emissions at the end of the period.

In the Netherlands (NL), the effect of energy intensity is rather intense in reducing emissions and, to a lesser extent, the energy mix term, which shows a smooth up-sloping trend. The activity term shows a constant increase, and the total emissions show a parabolic shape with a maximum around 2010 and a small net decrease at the end of the period.

In Poland (PO), the activity and the energy intensity contributions present almost identical trends; the first is positive, while the second has a negative slope, the population contribution is null all the time, and the effect of the energy mix is small and negative; therefore, the total emissions closely follow the energy mix trend, leading to a small reduction in emissions.

In Portugal (PT), the observed trends are similar to those of Greece and Italy, with the energy intensity and the energy mix contributions being positive at the beginning of the period and negative at the end; the population contribution is small and positive; the activity term increases rapidly until 2005 with a later constant trend; and the total emissions show a parabolic trend with a maximum around 2005 and a net reduction at the end of the period.

In Romania (RO), the energy intensity term is relatively strong and negative, while the population and energy intensity contributions are rather similar and negative but relatively modest. The activity contribution drops at the beginning of the period and then constantly increases, and finally, the total emissions continually decrease during the entire period.

In Slovakia (SK), the energy mix and energy intensity contributions are negative, the second is more negative, the population contribution is null, the activity contribution essentially has an up-sloping trend, and the total emission presents a continuous reduction during the entire period.

In Slovenia (SL), the energy mix and energy intensity terms present similar trends, being more negative than the second one; the effect of the population is positive but quite reduced; the activity term shows a rapid increase until 2008 with a later stabilization. Finally, the total emissions showed a parabolic trend with a small reduction at the end of the period.

In Spain (ES), its trend resembles that of Greece, Italy, and Portugal; the energy intensity and the energy mix contributions present an almost null contribution until 2005 and later a similar decrease; the activity contribution has a maximum around 2008 with later stabilization with oscillations; the population term shows a rapid increase until 2008 with a later stabilization. Finally, the total emissions show parabolic behavior with a maximum around 2008, with a tiny net increase at the end of the period.

In Sweden (SW), the energy mix and energy intensity contribution both have down-sloping trends, but stronger in the second case, the population contribution is up-sloping all the way with a sizable contribution, and the activity term shows an upward-sloping trend until 2008 with a later smaller increase. Finally, emissions showed a continuous decrease during the entire period.

In the European Union (EU) as a whole, the energy mix and energy intensity contributions show a very smooth and negative contribution, contributing more to the latter. The contribution of the population is small but always positive, with a particular slowdown in the last decade; the contribution of activity rapidly increases until 2008, with a later slowdown. Finally, the total emissions presented a relatively constant value until 2005, with a subsequent rapid decrease.

### 5.2 Tapio decoupling analysis

In the previous section, a common conclusion was that the activity term was the main cause of the increase in CO _2_ emissions. Therefore, it would be interesting to study the connection between CO _2_ emissions and GDP in more detail. The Tapio decoupling analysis is an ideal tool for exploring this connection.

**Figure 5.**
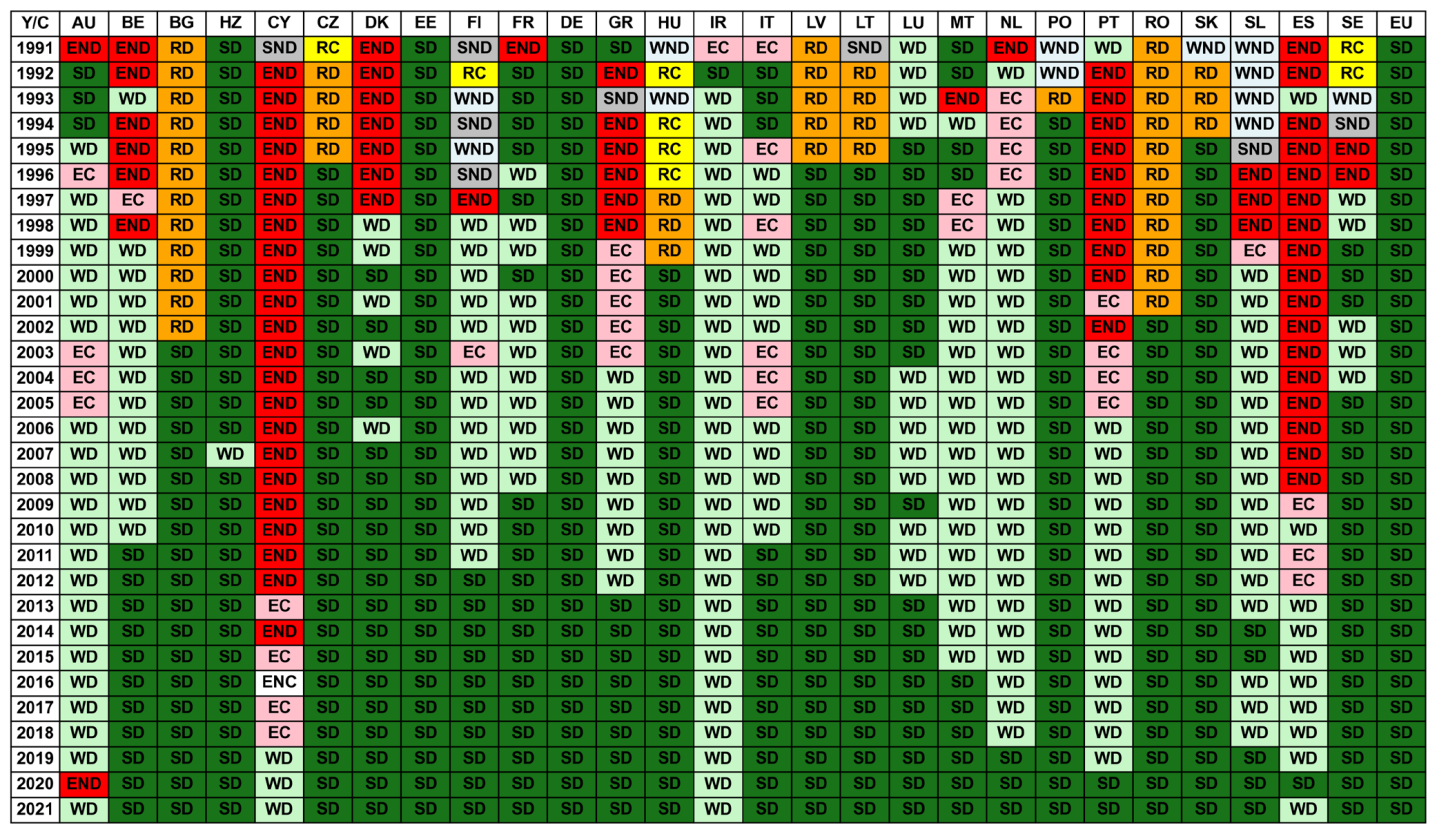
Time series of the Tapio decoupling analysis using the standard codes described in Figure 1. The green colours correspond to the more environmentally sustainable situations, while the red ones to the less environmentally sustainable ones.

We conducted a detailed analysis of the evolution of the Tapio coupling elasticity for all EU countries as a function of time within the period 1990–2021. In Figure 5, we present the time evolution of the different countries in terms of the Tapio class codes to which they belong. The net evolution of a major part of the country is self-evident in the Strong Decoupling (SD) or Weak Decoupling (WD) situation. This supposes that CO _2_ emissions reduce while GDP increases, or at least they increase in a reduced manner in all EU countries. Therefore, CO _2_ emissions started to decouple from GDP. However, to understand the decoupling level in detail, it is more convenient to present the time evolution in terms of emissions and GDP coordinates. This is shown in Figs. B1-B2 appearing in Appendix B in 55. Here, one can see a great variety of situations, even for countries that have moved into the Strong Decoupling region.

### 5.3 Convergence and cluster study

The convergence analysis enables the determination of whether European countries are moving toward similar emission levels using a rigorous statistical test. Owing to the varying sizes of countries, the analysis should always be conducted using relative rather than absolute levels. Specifically, multiplicative LMDI components were used (see Appendix A in 55). However, this analysis can be performed using raw or filtered data. In our case, the Hodrick-Prescott ^56^ filter is used. This filter allows the detection of outliers, such as economic crises or random market behavior, in the data under study. Thus, it is possible to obtain the trend component and perform more adequate estimations.

Table 1 presents a global convergence test for the LMDI components and total emissions using filtered data. For none of the components, a global convergence (for all countries) is reached, but partial convergence appears in clubs of countries that converge into common levels in the different components of CO _2_ emissions, including total emissions.

**Table 1.** Global convergence test in LMDI components (log(t) test) using filtered data.

Variable	$\hat{b}$ coefficient	SE	t-stat	Type of convergence
Population	-2.547	0.019	-135.767	Club
Activity	-1.191	0.075	-15.828	Club
Energy intensity	-1.471	0.035	-41.661	Club
Energy mix	-2.317	0.071	-32.649	Club
Total	-1.035	0.050	-20.811	Club

***Note***: Clubs were obtained using the algorithm recommended by Phillips and Sul ^17, 33^. The *t-stat* is the convergence test statistic, and it is a simple one-sided *t-test* with a critical value of *−*1 *.*65 (Phillips and Sul ^17, 33^). SE denotes standard error.

Table 2 shows the results of the club convergence analysis for the total emissions. Initially, four clubs were obtained, but later, it was proven that they could be reduced to three clubs plus a set of countries with divergent levels. The first club is formed by Austria, Belgium, Luxembourg, the Netherlands, Poland, Portugal, Slovenia, and Spain, and it converges to a total emissions level of 100 *−* 90% that of 1990. The second club was formed by the Czech Republic, Croatia, Finland, France, Germany, Greece, Hungary, Italy, Malta, and Sweden, which accounted for 80% of the emissions in 1990. The third club is made by Bulgaria, Denmark, Latvia, Lithuania, Romania, and Slovakia, and it converges to a level around 60 *−* 50% of that of 1990. Finally, the Cyprus, Estonia, and Ireland group diverges. We also obtained additional information from the value of the $\hat{b}$ /2 coefficient, which is related to the speed of convergence. Hence, Club 3 presents a faster convergence rate, while Club 2 is the slowest.

**Table 2.** Club convergence in total CO _2_ emissions using filtered data.

Club	No. of states	$\hat{b}$ coeff	SE	t-stat	States
1	8	0.209	0.171	1.222	Austria, Belgium, Luxembourg, Netherlands, Poland, Portugal, Slovenia, Spain
2	10	0.057	0.133	0.428	Czech Republic, Croatia, Finland, France, Germany, Greece, Hungary, Italy, Malta, Sweden
3	4	0.976	0.152	6.411	Bulgaria, Denmark, Lithuania, Slovakia.
4	2	2.336	0.268	8.701	Latvia, Romania
Divergent					Cyrpus, Estonia, Ireland
**Club merging algorithm**					
1	8	0.209	0.171	1.222	Austria, Belgium, Luxembourg, Netherlands, Poland, Portugal, Slovenia, Spain
2	10	0.057	0.133	0.428	Czech Republic, Croatia, Finland, France, Germany, Greece, Hungary, Italy, Malta, Sweden
3	6	0.495	0.133	3.722	Bulgaria, Denmark, Latvia, Lithuania, Romania, Slovakia
Divergent					Cyrpus, Estonia, Ireland

***Note***: Club merging assumes the null hypothesis that Clubs I and j can be considered in the same convergence club ^42^. The test is also distributed as a one-sided t-statistic with a 5% critical value of -1.65.

Appendix C in 55 presents a convergence analysis of the different LMDI components. In the case of population contribution (see table C1 in 55), seven clubs and a group of divergent countries can be established. The largest clubs were Italy, Greece, Slovenia, Germany, the Czech Republic, Slovakia, and Portugal, converging at a 1 *.*05 level. Concerning the activity contribution (see table C2), five clubs appear, but the largest one is made up of Bulgaria, Croatia, Czech Republic, Estonia, Hungary, Ireland, Latvia, Lithuania, Luxembourg, Poland, Malta, Netherlands, Romania, Slovakia, Slovenia, and Sweden, converging to a level in the range of 1 *.*6 *−* 2 *.*2. The case of energy intensity (see table C3) shows a large variety of trends, with eight existing convergence clubs and five diverging countries. Finally, for the energy mix contribution (see table C4), only two clubs exist plus two diverging countries, with the largest clubs made up of Austria, Belgium, Bulgaria, Croatia, Cyprus, France, Hungary, Ireland, Germany, Greece, Italy, Lithuania, Luxembourg, Malta, Netherlands, Poland, Portugal, Romania, Slovenia, and Spain, converging to a level of approximately 0 *.*8.

Convergence analysis focuses on the final points that evolve in different countries and explores only one component each time. Moreover, it does not consider a country’s global evolution over the entire period. Therefore, it would be interesting to compare countries’ global evolution, considering all LMDI components. It can be easily carried out by assigning to every country its entire time series corresponding to the four LMDI components (4 *×* 32 = 128 coordinates). Moreover, to represent the position of every country in the multidimensional space (128 dimensions), it is possible to perform a PCA, which defines a linear combination of the original variables corresponding to the largest variance. In our case, it was sufficient to consider the two principal components.

**Figure 6.**
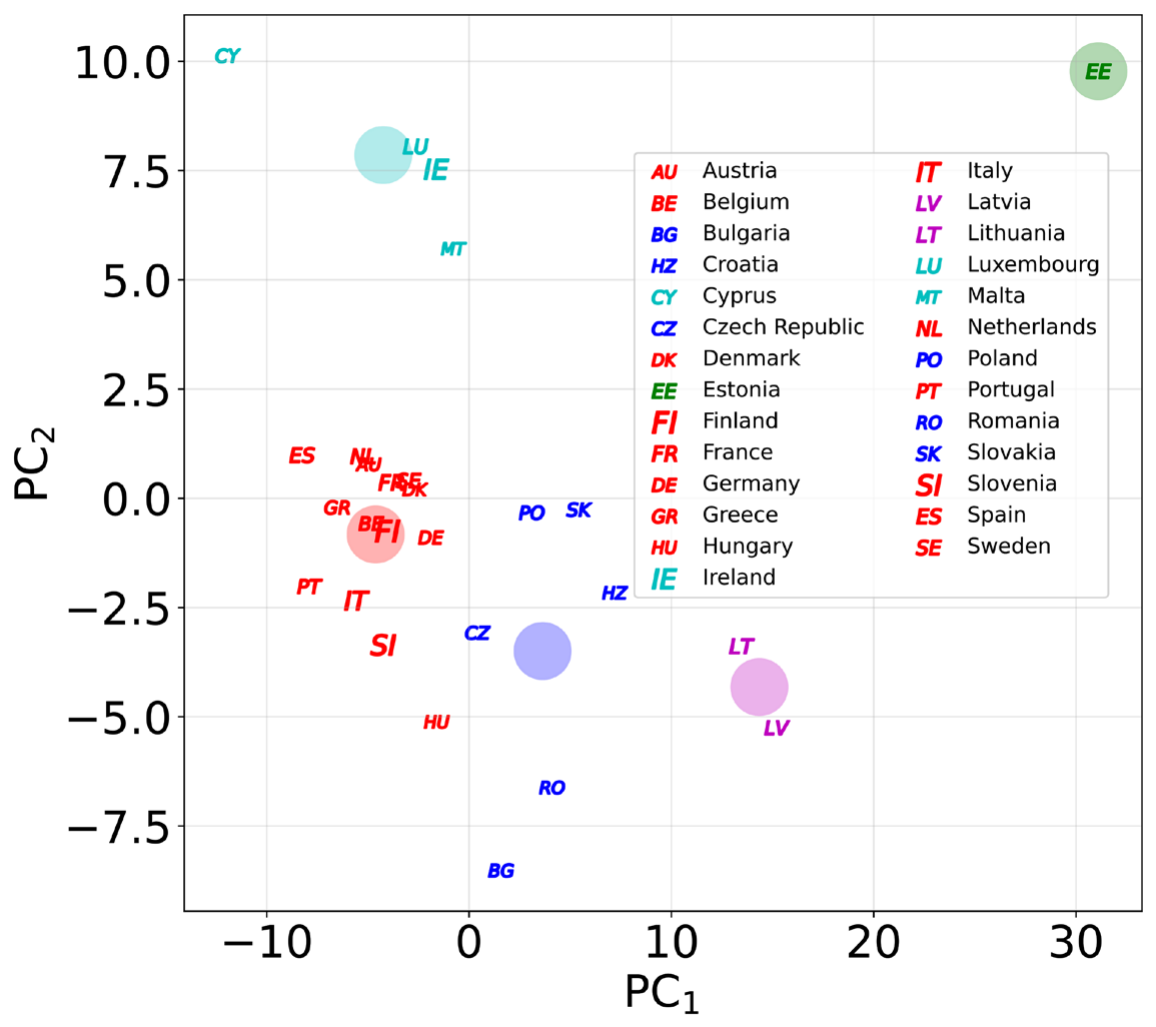
The position of the EU countries using two principal components for the LMDI time series. Different colours are used for different clusters, and the large circles correspond to the clusters’ centroids.

In Figure 6, we present the positions of all countries in terms of the first two principal components of the LMDI coordinates. Moreover, we used the K-means clustering method ^18^ to separate the groups with a similar trend in all components throughout the country. First, we analyzed the optimal number of clusters and found a value of 5. The analysis is also presented in Figure 6, which separates the clusters into colors. The obtained clusters are as follows:

- Cluster 1: Austria, Belgium, Denmark, Finland, France, Germany, Greece, Hungary, Italy, the Netherlands, Portugal, Slovenia, Spain, and Sweden.
- Cluster 2: Bulgaria, Croatia, Czech Republic, Poland, Romania, and Slovakia.
- Cluster 3: Cyprus, Ireland, Luxembourg, and Malta.
- Cluster 4: Latvia and Lithuania.
- Cluster 5: Estonia.

Cluster 1 corresponds to the countries that already belonged to the EU in 1995, after creating the European Economic Areas, except for Luxembourg and Ireland (also the UK), including Hungary. Therefore, they correspond to relatively large countries that have been part of the EU for many years and share common policies. Cluster 2 corresponds to a significant number of eastern countries that joined the EU in 2004 and onwards. Cluster 3 consists of small countries with similar fiscal structures and tax havens. Finally, clusters 4 and 5 correspond to Baltic Republics; two of them present similarities, but Estonia is clearly an outlier. In comparison with the convergence analysis, there was no clear overlap between the clubs and clusters. Cluster 1 overlaps with the sum of the countries from Clubs 1 and 2. Cluster 2 overlapped with Club 3. Finally, clusters 3, 4, and 5 overlapped with clubs 3 and 4 and the divergent group, respectively. In any case, a detailed overlap between both approaches does not exist because the criteria used to separate groups are quite different in the case of the convergence analysis compared with the cluster analysis.

### 5.4 Emission intensity

**Figure 7.**
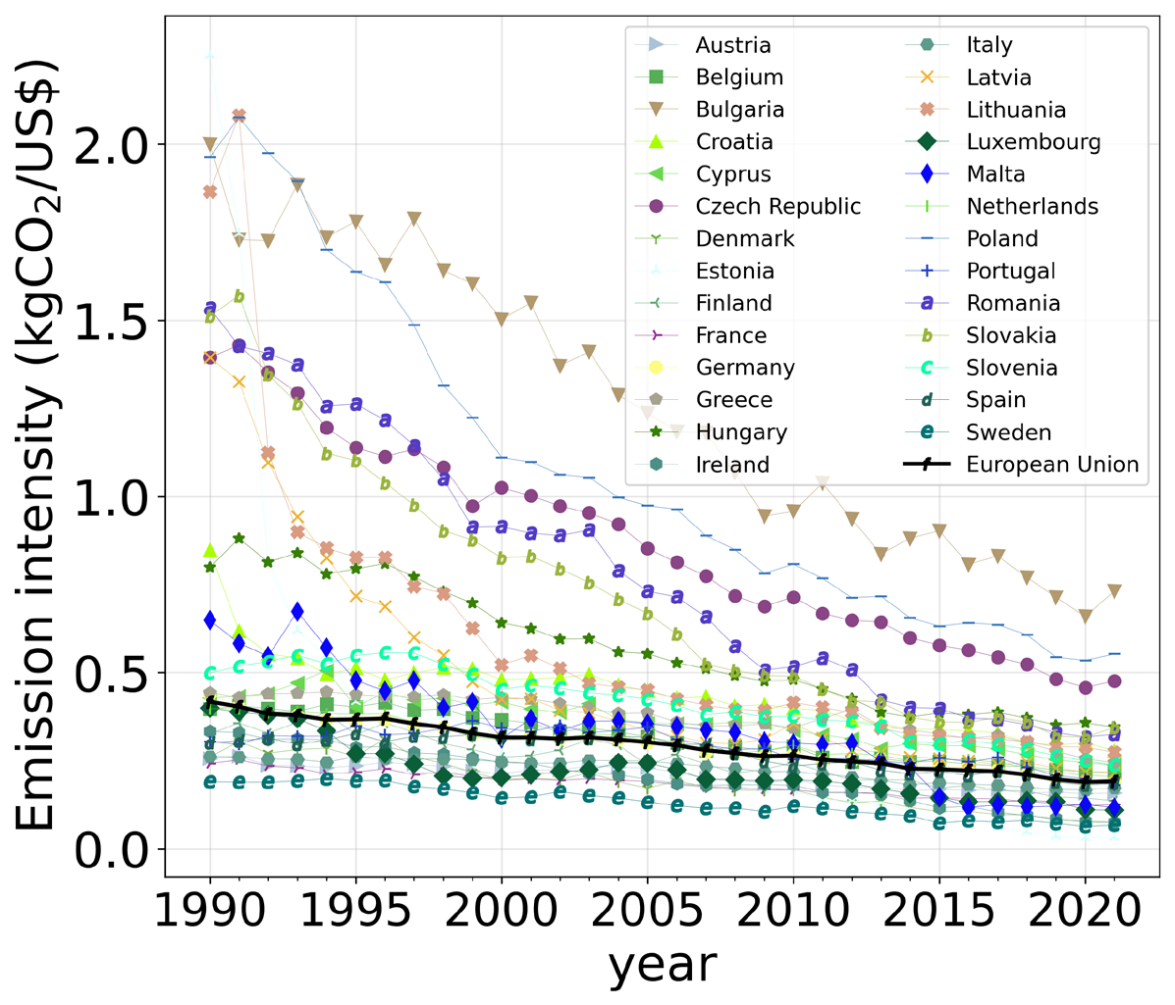
Evolution of the emission intensity for the countries of the EU.

A simple way to measure the performance of a given economy with respect to the reduction in CO _2_ emissions is emission intensity. According to Figure 7, the emission intensity of the EU is steadily declining, but presents a relatively constant value moving between 0 *.*4 and 0 *.*25 kgCO _2_/USD. However, there is a large number of countries that closely follow the EU trend, namely, Austria, Belgium, Cyprus, Denmark, Estonia, Finland, France, Germany, Greece, Ireland, Italy, Luxembourg, Netherlands, Portugal, Slovenia, Spain, and Sweden. A second group of countries, which is made of Bulgaria, Czech Republic, Latvia, Lithuania, Poland, Romania, and Slovakia, started with a huge value of emission intensity with a rapid drop, almost reaching the EU average, except Bulgaria, Poland, and the Czech Republic, which still have a relatively large value. Hungary started from an intermediate situation and still had an above-average value. Croatia also started from an intermediate situation, but it quickly reached the average value of the EU and, finally, Malta has reached an emission intensity value below the average, showing an abrupt transition in the last decade. It is worth noting that the present value for China and India is roughly 1 kgCO _2_/USD, which is larger than the value for the worst of the European countries, Bulgaria ^12^. Moreover, as in China, the European trend is downsloping, whereas a certain stabilization has been observed in India. The USA presents a trend similar to that of the EU.

## 6. Summary, conclusions and policy implication

In this work, we have studied the driving forces of the CO _2_ emissions of the 27 countries of the European Union to obtain a detailed analysis of the evolution of CO _2_ emissions from 1990 to 2021 for all their members and, at the same time, find possible clubs of convergence, clusters, and similarities between these countries. First, we considered the Kaya identity, where emissions are connected with population, GDP per capita, energy intensity, and energy matrix. Note that owing to the large number of countries considered, we have decided to simplify the analysis by considering only four types of fuel sources: solid fossil, liquid fossil, gas fossil, and renewable energy. Moreover, no disaggregation between economic sectors was used. The analysis was conducted using the LMDI method to separate the contributions of different driving forces. Then, Tapio decoupling analysis was performed to determine how tightly connected CO _2_ emissions and GDP are. Next, we conducted a convergence analysis complemented by a cluster study to separate the different countries into groups that present similarities in their time evolution.

Section 5.1 presents the time evolution of the additive LMDI decomposition for all EU countries (see Figure 2 and Figure 3), and in Appendix A in 55, the multiplicative LMDI decomposition is also provided (see Figs. A1 and A2 in 55). This detailed set of figures provides a clear view of the evolution of CO _2_ emissions in every country in the EU. This allows a comparison among them, which helps to draw quick conclusions. The first conclusion is that the main driving force favoring CO _2_ emissions is the activity term, which is strongly related to economic development.

To go a step further in our understanding of this connection, we also explored the Tapio decoupling analysis (see Section 5.2 and Appendix B in 55), concluding that the majority of countries are moving into a strong decoupling between CO _2_ emissions and GDP (see Figs. B1 and B2 in 55). Regarding the contributors to the reduction in CO _2_ emissions, energy intensity is the strongest, which is a common feature in all countries. The second is the energy mix term, which is not so intense so far, and its trend depends strongly on the country. Finally, population is a minor contributor, although it is positive in most countries.

Considering the EU total results, one can notice that its global emissions have been reduced by 26% with respect to 1990, which is in line with the fulfilment of the EU NDC. Contributing to this number, the activity term increases by 51% and the population term by 6%, while the mixing term presents a reduction of 24% and an energy intensity term of 40%.

The following step has sought common features between the evolution of the driving forces of CO _2_ emissions in all EU member states over the past three decades. In order to group the countries in ensembles with similar behavior, we performed a convergence analysis, which allowed us to separate the countries according to their final level of emissions. Different convergence clubs can be defined for total emissions. The first club, formed by Austria, Belgium, Luxembourg, the Netherlands, Poland, Portugal, Slovenia, and Spain, converged to a level of total emissions of 100 *−* 90% of those of 1990. The second club, formed by Croatia, the Czech Republic, Finland, France, Germany, Greece, Hungary, Italy, Malta, and Sweden, moved to 80% of the emissions in 1990. The third club, made up of Bulgaria, Denmark, Lithuania, Latvia, Romania, and Slovakia, converged to a level around 60 *−*50% that of 1990. Finally, the group of Cyprus, Estonia, and Ireland diverged.

Moreover, a cluster analysis was performed on all LMDI components in different countries. The main difference with the convergence analysis is that in this case, not only the final value of the emission level but also the entire evolution is important. Cluster analysis allows for separation into five groups that have certain overlaps with the clubs. The structure of the different clusters can be easily understood because they are directly connected with the year of incorporation of the countries into the EU and, therefore, with the moment they started implementing EU policies regarding CO _2_ emissions. The first cluster includes Austria, Belgium, Denmark, Finland, France, Germany, Greece, Hungary, Italy, the Netherlands, Portugal, Slovenia, Spain, and Sweden. Most countries that have belonged to the EU since the 1990s have shared common policies for an extended period. The second cluster is formed by Bulgaria, Croatia, the Czech Republic, Poland, Romania, and Slovakia, corresponding to the majority of eastern countries that joined the EU from 2004 onwards. The third cluster comprises Cyprus, Ireland, Luxembourg, and Malta, which are small countries in terms of size and population, and they offer very similar corporate tax treatment and incentives. Finally, Clusters 4 and 5 correspond to the Baltic Republics of Estonia, Latvia, and Lithuania.

Overall, the EU shows an evident decline in its CO _2_ emissions, and it is on the way to reach the target of a 55% reduction in 2030, as established in its last NDC, which will be an excellent achievement for EU Climate Change policy. In the previous sections, we observed that different countries show different paces in CO _2_ reductions. Usually, those countries that have belonged to the EU for a long time more often present better behavior, which could be a extra proof in favor of the EU policy.

As already mentioned, the EU is the third largest global emitter and will most probably be passed by India in the coming years, but it is very far from China’s emissions level (three times that of the EU). Therefore, the case of the EU is not only noticeable because of the net reduction of emissions, but also because this region is developing technologies and new policies that prove that it is possible to have a significant reduction in CO _2_ emissions even under a continuous increase in GDP during long periods. In other words, the EU can be considered a successful testbed for Climate Change policies and technology. Therefore, it is highly recommended that these good practices and policies be exported to other countries, especially developing countries.

## Ethics and consent

Ethical approval and consent were not required.

## Data Availability

Zenodo: Underlying data for “European Union data: GDP and energy in the period 1990–2021”. https://doi.org/10.5281/zenodo.15220467 ^57^. The project contains the following underlying data: DatosLDMI2-bis.xls: raw data used in the calculations Zenodo: SUPPLEMENTARY MATERIAL for “Exploring the driving forces of CO _2_ emissions in the European Union”. https://doi.org/10.5281/zenodo.15293907 ^55^. Data are available under the terms of the Creative Commons Attribution 4.0 International.

## References

[1] Stocker TF Qin D Plattner GK : Climate change 2013: the physical science basis. Contribution of working group I to the fifth assessment report of the intergovernmental panel on climate change. Cambridge University Press, Cambridge, United Kingdom and New York, NY, USA,2013. Reference Source

[2] Masson-Delmotte V Zhai P Pörtner HO : Summary for policymakers.In: *Global Warming of 1.5° C. An IPCC Special Report on the impacts of global warming of 1.5° C above pre-industrial levels and related global greenhouse gas emission pathways, in the context of strengthening the global response to the threat of climate change, sustainable development, and efforts to eradicate poverty*. World Meteorological Organization, Geneva, Switzerland,2018. Reference Source

[3] EPA (United States Environmental Protection Agency): Global greenhouse gas emissions data.2024; Accessed date: 1 December 2024. Reference Source

[4] Kaya Y : Impact of carbon dioxide emission control on GNP growth: interpretation of proposed scenarios. Paper presented to the IPCC Energy and Industry Subgroup, Response Strategies Working Group, Paris, (mimeo).1990.

[5] Kaya Y Yokobori K : Environment, energy, and economy: strategies for sustainability. *Conference on Global Environment, Energy, and Economic Development (1993 : Tokyo, Japan)*,1993.

[6] Kaya Y Yokobori K : Environment, energy and economy; strategies for sustainability. United Nations University Press,1997. Reference Source

[7] United Nations Framework Convention on Climate Change (UNFCCC): Nationally determined contributions.2024; Accessed date: 1 December 2024. Reference Source

[8] Robalino-López A García-Ramos JE Mena-Nieto A : System dynamics modelling and the environmental Kuznets curve in Ecuador (1980–2025). *Energy Policy.* 2014;67:923–931. 10.1016/j.enpol.2013.12.003

[9] Robalino-López A Mena-Nieto A García-Ramos JE : System dynamics modeling for renewable energy and CO _2_ emissions: a case study of Ecuador. *Energy Sustain Dev.* 2014;20:11–20. 10.1016/j.esd.2014.02.001

[10] Robalino-López A Mena-Nieto A García-Ramos JE : Studying the relationship between economic growth, CO _2_ emissions, and the environmental Kuznets curve in Venezuela (1980-2025). *Renew Sust Energ.* 2015;41:602–614. 10.1016/j.rser.2014.08.081

[11] Ortega-Ruiz G Mena-Nieto A García-Ramos JE : Is India on the right pathway to reduce CO _2_ emissions? Decomposing an enlarged Kaya identity using the LMDI method for the period 1990-2016. *Sci Total Environ.* 2020;737: 139638. 10.1016/j.scitotenv.2020.139638 32512297

[12] Ortega-Ruiz G Mena-Nieto A Golpe AA : CO _2_ emissions and causal relationships in the six largest world emitters. *Renew Sustain Energy Rev.* 2022;162: 112435. 10.1016/j.rser.2022.112435

[13] Borja-Patiño J Robalino-López A Mena-Nieto A : Breaking the unsustainable paradigm: exploring the relationship between energy consumption, economic development and carbon dioxide emissions in Ecuador. *Sustainability Science.* 2024;19(2):403–421. 10.1007/s11625-023-01425-x

[14] Ang BW Choi KH : Decomposition of aggregate energy and gas emission intensities for industry: a refined divisia index method. *Energy J.* 1997;18(3):59–73. Reference Source

[15] Tapio P : Towards a theory of decoupling: degrees of decoupling in the EU and the case of road traffic in Finland between 1970 and 2001. *Transp Policy.* 2005;12(2):137–151. 10.1016/j.tranpol.2005.01.001

[16] Wang W Liu X Zhang M : Using a new generalized LMDI (Logarithmic Mean Divisia Index) method to analyze China’s energy consumption. *Energy.* 2014;67:617–622. 10.1016/j.energy.2013.12.064

[17] Phillips PCB Sul D : Transition modeling and econometric convergence tests. *Econometrica.* 2007;75(6):1771–1855. 10.1111/j.1468-0262.2007.00811.x

[18] Lloyd S : Least squares quantization in PCM. *IEEE Trans Inf Theory.* 1982;28(2):129–137. Reference Source

[19] Marcucci A Fragkos P : Drivers of regional decarbonization through 2100: a multi-model decomposition analysis. *Energy Econ.* 2015;51:111–124. 10.1016/j.eneco.2015.06.009

[20] Fernández-González P Landajo M Presno MJ : The driving forces behind changes in CO _2_ emission levels in EU-27. Differences between member states. *Environ Sci Policy.* 2014;38:11–16. 10.1016/j.envsci.2013.10.007

[21] Fernández-González P Landajo M Presno MJ : Tracking European Union CO _2_ emissions through LMDI (Logarithmic-Mean Divisia Index) decomposition. The activity revaluation approach. *Energy.* 2014;73:741–750. 10.1016/j.energy.2014.06.078

[22] Madaleno M Moutinho V : A new LDMI decomposition approach to explain emission development in the EU: individual and set contribution. *Environ Sci Pollut Res Int.* 2017;24(11):10234–10257. 10.1007/s11356-017-8547-y 28265876

[23] Moutinho V Madaleno M Silva PM : Which factors drive CO _2_ emissions in EU-15? Decomposition and innovative accounting. *Energy Effic.* 2016;9:1087–1113. 10.1007/s12053-015-9411-x

[24] Karmellos D Kopidou M Diakoulaki D : Decomposition analysis of the driving factors of CO _2_ (Carbon dioxide) emissions from the power sector in the European Union countries. *Energy.* 2016;94:680–692. 10.1016/j.energy.2015.10.145

[25] Liobikiené G Butkus M Bernatoniené J : Drivers of greenhouse gas emissions in the Baltic states: decomposition analysis related to the implementation of Europe 2020 strategy. *Renew Sustain Energy Rev.* 2016;54:309–317. 10.1016/j.rser.2015.10.028

[26] Balsalobre-Lorente D Shahbaz M Roubaud D : How economic growth, renewable electricity and natural resources contribute to CO _2_ emissions? *Energy Policy.* 2018;113:356–367. 10.1016/j.enpol.2017.10.050

[27] Dogan E Aslan A : Exploring the relationship among CO _2_ emissions, real GDP, energy consumption and tourism in the EU and candidate countries: evidence from panel models robust to heterogeneity and cross-sectional dependence. *Renew Sustain Energy Rev.* 2017;77:239–245. 10.1016/j.rser.2017.03.111

[28] Cruz L Dias J : Energy and CO _2_ intensity changes in the EU-27: decomposition into explanatory effects. *Sustain Cities Soc.* 2016;26:486–495. 10.1016/j.scs.2016.03.007

[29] Hatzigeorgiou E Polatidis H Haralambopoulos D : CO _2_ emissions in Greece for 1990–2002: A decomposition analysis and comparison of results using the Arithmetic Mean Divisia Index and Logarithmic Mean Divisia Index techniques. *Energy.* 2008;33(3):492–499. 10.1016/j.energy.2007.09.014

[30] Karmellos M Kosmadakis V Dimas P : A decomposition and decoupling analysis of carbon dioxide emissions from electricity generation: evidence from the EU-27 and the UK. *Energy.* 2021;231: 120861. 10.1016/j.energy.2021.120861

[31] Papiez M Smiech S Frodyma K : The role of energy policy on the decoupling processes in the European Union countries. *J Clean Prod.* 2021;318: 128484. 10.1016/j.jclepro.2021.128484

[32] Bianco V Cascetta F Nardini S : Analysis of the carbon emissions trend in European Union. A decomposition and decoupling approach. *Sci Total Environ.* 2024;909: 168528. 10.1016/j.scitotenv.2023.168528 37963528

[33] Phillips PCB Sul D : Economic transition and growth. *J Appl Econ.* 2009;24(7):1153–1185. 10.1002/jae.1080

[34] Robalino-López A García-Ramos JE Golpe AA : CO _2_ emissions convergence among 10 South American countries. A study of Kaya components (1980–2010). *Carbon Manag.* 2016;7(1–2):1–12. 10.1080/17583004.2016.1151502

[35] Jobert T Karanfil F Tykhonenko A : Convergence of per capita carbon dioxide emissions in the EU: legend or reality? *Energy Economics.* 2010;32(6):1364–1373. 10.1016/j.eneco.2010.03.005

[36] Camarero M Picazo-Tadeo AJ Tamarit C : Are the determinants of CO _2_ emissions converging among OECD countries? *Econ Lett.* 2013;118(1):159–162. 10.1016/j.econlet.2012.10.009

[37] Rodríguez-Benavides D Andrés-Rosales R Álvarez García J : Convergence of clubs between per capita carbon dioxide emissions from fossil fuels and cement production. *Energy Policy.* 2024;186: 114007. 10.1016/j.enpol.2024.114007

[38] Camarero M Castillo-Giménez J Picazo-Tadeo AJ : Is eco-efficiency in greenhouse gas emissions converging among European Union countries? *Empirical Economics.* 2014;47:143–168. 10.1007/s00181-013-0734-1

[39] Morales-Lage R Bengochea-Morancho A Camarero M : Club convergence of sectoral CO _2_ emissions in the European Union. *Energy Policy.* 2019;135: 111019. 10.1016/j.enpol.2019.111019

[40] Cialani C Mortazavi R : Sectoral analysis of club convergence in EU countries’ CO _2_ emissions. *Energy.* 2021;235: 121332. 10.1016/j.energy.2021.121332

[41] Emir F Balcilar M Shahbaz M : Inequality in carbon intensity in EU-28: analysis based on club convergence. *Environ Sci Pollut Res Int.* 2019;26(4):3308–3319. 10.1007/s11356-018-3858-1 30506441

[42] Camarero M Castillo J Picazo-Tadeo AJ : Eco-efficiency and convergence in OECD countries. *Environ Resour Econ.* 2013;55(1):87–106. 10.1007/s10640-012-9616-9

[43] Presno MJ Landajo M : EU-28’s progress toward the 2020 renewable energy share: a club convergence analysis. *Environ Sci Pollut Res Int.* 2021;28(47):66830–66844. 10.1007/s11356-021-15084-x 34236612 PMC8265289

[44] World Bank: Data and statistics for the European Union. Accessed date: 1 December 2024,2024. Reference Source

[45] Eurostat: Statistics explained: development of the production of primary energy (by fuel type). Accessed date: 1 December 2023,2023. Reference Source

[46] Ang BW Zhang FQ Choi KH : Factorizing changes in energy and environmental indicators through decomposition. *Energy.* 1998;23(6):489–495. 10.1016/S0360-5442(98)00016-4

[47] Ang BW Liu FL : A new energy decomposition method: Perfect in decomposition and consistent in aggregation. *Energy.* 2001;26(6):537–547. Reference Source

[48] Ang BW : Decomposition analysis for policymaking in energy: which is the preferred method? *Energy Policy.* 2004;32(9):1131–1139. 10.1016/S0301-4215(03)00076-4

[49] Ang BW : The LMDI approach to decomposition analysis: a practical guide. *Energy Policy.* 2005;33(7):867–871. 10.1016/j.enpol.2003.10.010

[50] Ang BW : LMDI decomposition approach: a guide for implementation. *Energy Policy.* 2015;86:233–238. 10.1016/j.enpol.2015.07.007

[51] Tapio P Banister D Luukkanen J : Energy and transport in comparison: Immaterialisation, dematerialisation and decarbonisation in the EU15 between 1970 and 2000. *Energy Policy.* 2007;35:433–451. Reference Source

[52] Jolliffe IT Cadima J : Principal component analysis: a review and recent developments. *Phil Trans R Soc A.* 2016;374(2065): 20150202. 10.1098/rsta.2015.0202 26953178 PMC4792409

[53] IPCC (Intergovernmental Panel on Climate Change): Intergovernmental panel on climate change data. Accessed date: 1 December 2024,2024. Reference Source

[54] IPCC (Intergovernmental Panel on Climate-Change): 2006 IPCC Guidelines for National Greenhouse Gas Inventories, Prepared by the National Greenhouse Gas Inventories Programme.Cambridge University Press,2006. Reference Source

[55] García-Ramos JE : European Union data: GDP and energy in the period 1990–2021. [Data set]. Zenodo,2025. 10.5281/zenodo.15220468

[56] Hodrick R Prescott E : Postwar U.S. business cycles: an empirical investigation. Carnegie-Mellon University; Discussion Papers 451, Northwestern University,1980.

[57] Cámara-Aceituno J Hermoso-Orzáez MJ Terrados-Cepeda J : Supplementary Material for Exploring the driving forces of CO _2_ emissions in the European Union. [Data set]. Zenodo,2025. 10.5281/zenodo.15293907 PMC12134738 40469273

