# Supplementary material for: Exploring the driving forces of CO 2 emissions in the European Union
Source: Open Res Eur. 2025 May 12;5:132. [Version 1] doi: 10.12688/openreseurope.20224.1 (PMC12134738; doi:10.12688/openreseurope.20224.1)
Supplement: Peer review history

# Reviewer report: Reviewer response for version 1

**DOI:** 10.21956/openreseurope.21884.r54472

**Published:** 2025-06-03

**Authors:** Matheus Koengkan

**Affiliations:** 1 University of Coimbra, Coimbra, Portugal

- Reviewer comment (R.C.): While the paper mentions that a comprehensive approach combining five methods has not been published before, the novelty claim should be clarified and justified with stronger differentiation from recent, similar multi-method EU studies.
- Reviewer comment (R.C.): The abstract is overly dense with methodology and results. Consider restructuring it to more clearly distinguish the objective, method, key results, and conclusions.
- Reviewer comment (R.C.): The current title is broad. Consider adding a hint of the methodology used (e.g., LMDI, Tapio) or time span (1990–2021) to better reflect the study’s scope.
- Reviewer comment (R.C.): The manuscript attempts to do a lot in one paper—LMDI, Tapio, convergence, cluster, and PCA analyses. Consider whether some sections could be streamlined or deferred to supplementary materials for clarity.
- Reviewer comment (R.C.): The paper assumes fixed emission factors (e.g., for fossil fuels), which may have changed due to improved combustion efficiency or technological advances. A sensitivity analysis would strengthen the validity of results.
- Reviewer comment (R.C.): The omission of sectoral disaggregation (e.g., transport, industry, households) is a missed opportunity and should at least be acknowledged as a limitation.
- Reviewer comment (R.C.): There is no discussion of uncertainty or variability in the input data or modeling assumptions. Adding confidence intervals or sensitivity checks would increase robustness.
- Reviewer comment (R.C.): The use of constant 2010 USD is standard, but the source and justification for deflators should be clearly stated.
- Reviewer comment (R.C.): Why does the analysis start in 1990 and end in 2021? The COVID-19 years have anomalous trends—should they be treated differently or modeled separately?
- Reviewer comment (R.C.): The K-means algorithm has well-known limitations (e.g., sensitivity to initial centroids, scale dependency). Justify its use over more robust methods like DBSCAN or hierarchical clustering.
- Reviewer comment (R.C.): While Phillips and Sul’s method is used for convergence, there is no validation or comparison with alternative clustering or convergence detection techniques.
- Reviewer comment (R.C.): More explanation is needed on how data were normalized for clustering and PCA. This step significantly affects the results and should not be glossed over.
- Reviewer comment (R.C.): Was any test for multicollinearity performed before applying PCA? The assumption of orthogonality in components needs to be validated.
- Reviewer comment (R.C.): The Zenodo data repository is mentioned, but the DOI link is broken. Please ensure full accessibility of the underlying dataset.
- Reviewer comment (R.C.): Several figures (e.g., Figure 3–4 and Figure 6) are overcrowded and hard to interpret. Consider moving country-level LMDI decompositions to supplementary materials.
- Reviewer comment (R.C.): The use of colors in Tapio and PCA plots needs improvement for accessibility. Consider using patterns or markers to distinguish lines for colorblind readers.
- Reviewer comment (R.C.): The manuscript is overwhelmed by acronyms, some of which are defined too late or used inconsistently (e.g., LMDI, PC, EI). A glossary or acronym list would help.
- Reviewer comment (R.C.): Table 1 and Table 2 are not discussed in sufficient depth. The narrative should clearly explain what each club implies and why it matters.
- Reviewer comment (R.C.): References to "Appendix A in 55" are confusing and ambiguous. Ensure clear, consistent labeling of supplementary files or appendices.
- Reviewer comment (R.C.): For a study spanning 32 years, trend lines or smoothing in key figures (e.g., emissions intensity) would be more informative than raw bar plots.
- Reviewer comment (R.C.): The literature review section is extensive but lacks synthesis. Rather than listing 10+ similar studies, categorize them and explain how this study fills a specific gap.
- Reviewer comment (R.C.): The discussion mentions EU policies but is vague on specific regulations (e.g., ETS, Green Deal). Consider integrating actual policy milestones into the analysis.
- Reviewer comment (R.C.): While comparisons with the US and China are made, the manuscript would benefit from a short subsection explicitly situating the EU’s progress in a global context.
- Reviewer comment (R.C.): Several key 2023–2024 studies using hybrid LMDI and convergence methods in the EU are missing. Update citations to reflect the most recent developments.
- Reviewer comment (R.C.): The paper risks suggesting causal relationships between drivers and emissions without robust econometric backing. Consider revising language to focus on correlations.
- Reviewer comment (R.C.): The assertion that “longer-standing EU members perform better” is too broad. Are there notable exceptions? What about countries with different industrial bases?
- Reviewer comment (R.C.): The 2008 financial crisis and COVID-19 pandemic are mentioned but not integrated into the model interpretation. A brief “shock-response” subsection would be valuable.
- Reviewer comment (R.C.): A missed opportunity is the lack of targeted policy recommendations for each convergence club or cluster. This would make the study more actionable for policymakers.
- Reviewer comment (R.C.): The discussion of emission intensity trends is largely descriptive. Explain why some countries (e.g., Bulgaria, Poland) remain above the EU average and what that implies.
- Reviewer comment (R.C.): While the manuscript is readable, it contains multiple long and overly technical sentences. Proofreading for conciseness, grammar, and flow is recommended throughout.

If applicable, is the statistical analysis and its interpretation appropriate?

Yes

Is the study design appropriate and is the work technically sound?

Yes

Is the work clearly and accurately presented and does it engage with the current literature?

Yes

Are the conclusions drawn adequately supported by the results?

Yes

Are sufficient details of methods and analysis provided to allow replication by others?

Yes

Are all the source data and materials underlying the results available?

Yes

Reviewer Expertise:

The study presents valuable contributions but has some weaknesses that limit its full academic merit in the current form. These include issues such as unclear novelty claims, methodological assumptions needing clarification or sensitivity checks, and some presentation aspects that reduce clarity. However, these concerns can be addressed through targeted revisions, which would enhance the manuscript’s rigor and clarity without requiring a fundamental redesign.

I confirm that I have read this submission and believe that I have an appropriate level of expertise to confirm that it is of an acceptable scientific standard, however I have significant reservations, as outlined above.

# Reviewer report: Reviewer response for version 1

**DOI:** 10.21956/openreseurope.21884.r54229

**Published:** 2025-06-02

**Authors:** Vincenzo Bianco

**Affiliations:** 1 Universita degli Studi di Napoli Parthenope, Naples, Campania, Italy

The proposed paper is very interesting and informative. It offers a detailed analysis on the evolution of CO2 emissions in EU countries. Authors propose the classical formulation of the Kaya identity, complemented by the Tapio methodology for decoupling analysis and by an interesting cluster and convergence analysis. In the following some specific advices for a possible further improvement of the paper:

 1- Authors should specify the units of measure of the parameters included in the Kaya identity. In particular, authors should also specify if GDP is expressed in nominal or real values (real values should be considered).

 2- Convergence and Cluster analysis may be illustrated in a more specific way for the analysis developed in the paper rather than in general terms as is now.

 3- In the analysis of the results of LMDI, a discussion comparing the different countries.

 However, the provided comments are an indication to improve the paper, but the work is appreciable as is.

If applicable, is the statistical analysis and its interpretation appropriate?

Yes

Is the study design appropriate and is the work technically sound?

Yes

Is the work clearly and accurately presented and does it engage with the current literature?

Yes

Are the conclusions drawn adequately supported by the results?

Yes

Are sufficient details of methods and analysis provided to allow replication by others?

Yes

Are all the source data and materials underlying the results available?

Yes

Reviewer Expertise:

Energy systems; energy efficiency; sustainability; energy modelling

I confirm that I have read this submission and believe that I have an appropriate level of expertise to confirm that it is of an acceptable scientific standard.

# Reviewer report: Reviewer response for version 1

**DOI:** 10.21956/openreseurope.21884.r54473

**Published:** 2025-05-29

**Authors:** Bojan Pejovic

**Affiliations:** 1 University of Montenegro, Podgorica, Montenegro

The analysis can be strengthened by incorporating the Environmental Kuznets Curve (EKC), which suggests an inverted U-shaped link between income and environmental degradation. This extension would allow for a deeper understanding of how economic growth initially worsens, then improves environmental outcomes. Empirically, EKC can be tested using data on emissions or resource use versus GDP per capita, offering policy insights on when and how sustainability improves with development.

If applicable, is the statistical analysis and its interpretation appropriate?

Yes

Is the study design appropriate and is the work technically sound?

Yes

Is the work clearly and accurately presented and does it engage with the current literature?

Yes

Are the conclusions drawn adequately supported by the results?

Yes

Are sufficient details of methods and analysis provided to allow replication by others?

Yes

Are all the source data and materials underlying the results available?

Yes

Reviewer Expertise:

The paper is very good and provides a solid foundation for analyzing the relationship between economic development and the environment. A further theoretical or empirical extension using the Environmental Kuznets Curve (EKC)—which suggests an inverted U-shaped relationship between income per capita and environmental degradation—would significantly enhance the analysis. With such an addition, the paper has strong potential for publication in a relevant academic journal.4o

I confirm that I have read this submission and believe that I have an appropriate level of expertise to confirm that it is of an acceptable scientific standard, however I have significant reservations, as outlined above.
